# Heterotopic sebaceous glands in the esophagus, a very rare histopathological diagnosis: a case report and review of the literature

**DOI:** 10.1002/ccr3.791

**Published:** 2016-12-29

**Authors:** Nelson Montalvo, Verónica Tapia, Hernán Padilla, Ligia Redrobán

**Affiliations:** ^1^ Servicio de PatologíaHospital MetropolitanoQuitoEcuador; ^2^ Departamento de PatologíaHospital de Sociedad de Lucha contra el Cáncer [SOLCA]QuitoEcuador; ^3^ Servicio de EndoscopíaHospital de Clínicas PichinchaQuitoEcuador

**Keywords:** Endoscopy, esophagus, gastroesophageal reflux, heterotopic sebaceous glands

## Abstract

Sebaceous glands are very rarely found in the esophagus. Existing reports do not contain sufficient epidemiological, etiological, clinical, or prognostic data. Its histogenesis suggests heterotopia or metaplasia. Despite its extreme rarity, correct and generally easy identification enables establishing the proper patient monitoring.

## Introduction

Sebaceous glands are very rarely found in the esophagus. Existing reports do not contain sufficient epidemiological, etiological, clinical, or prognostic data. Its histogenesis suggests heterotopia or metaplasia 1. Despite its extreme rarity, correct and generally easy identification enables establishing the proper patient monitoring.

## Case Presentation

**Figure 1:**
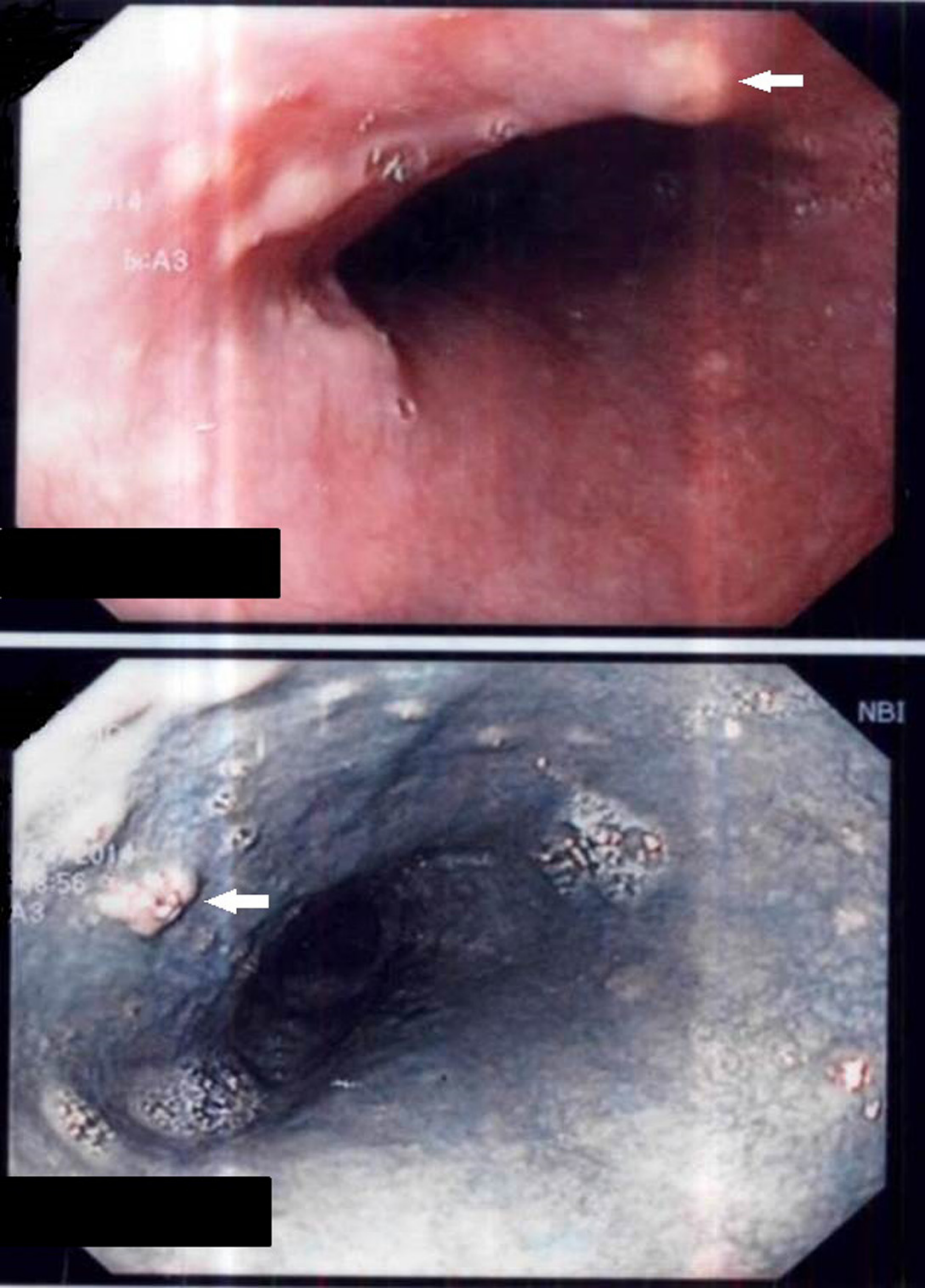
Endoscopy findings in the esophagus: several irregular yellowish lesions on the surface of the squamous mucosa [arrows]. The lower picture corresponds to a NBI image.

**Figure 2:**
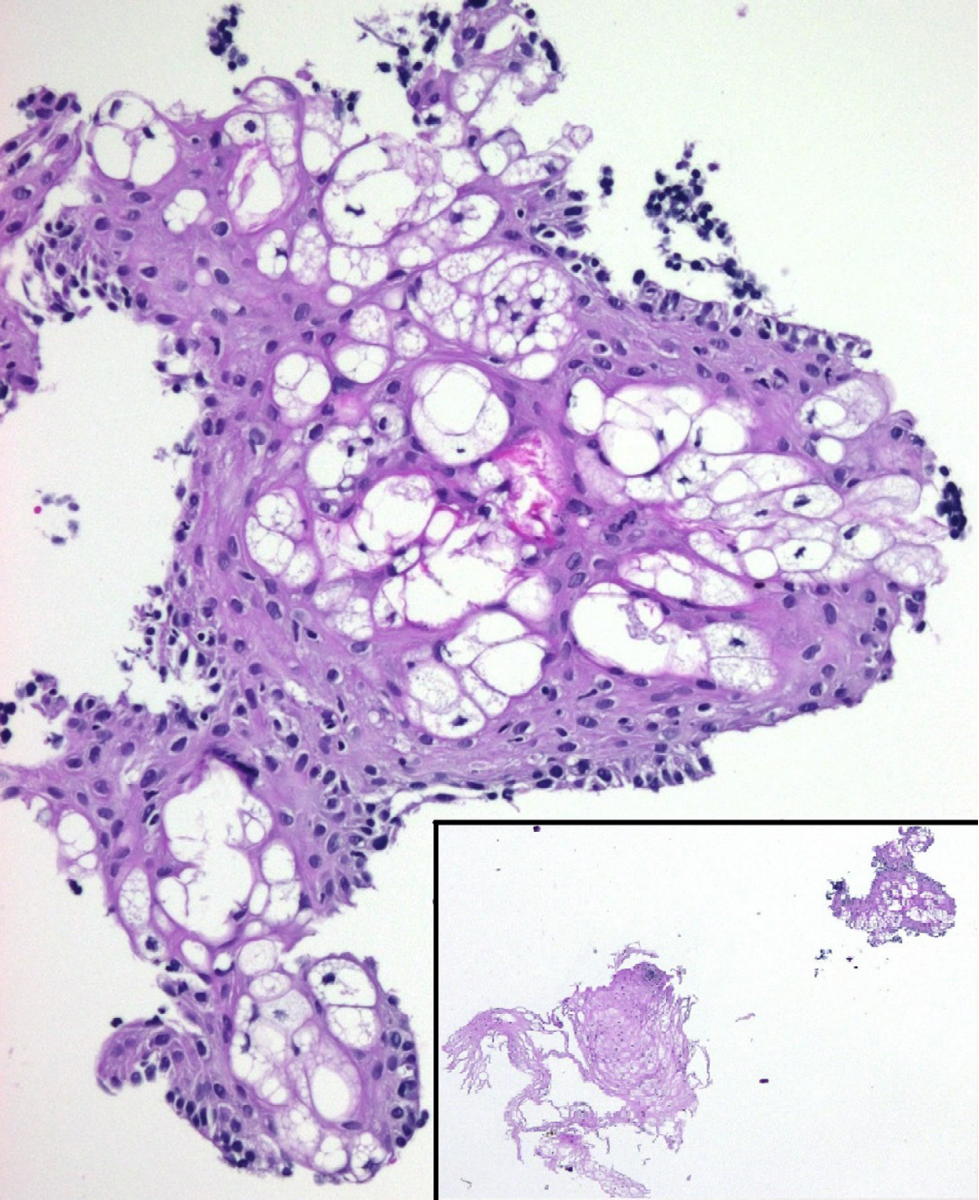
Heterotopic sebaceous glands: Large and polygonal, clear cell with vacuolated cytoplasm is seen here within the squamous epithelium [H/E 40 x [inset 4 x]].

We report the case of a 49‐year‐old symptomatic male with a 20‐year history of gastroesophageal reflux [GERD], consisting of epigastric burning and heartburn episodes of exacerbation and symptomatic treatment with proton pump inhibitors [esomeprazole 40 mg QD]. The patient took the drug irregularly. An endoscopic study 4 years previously had shown no lesions. A later esophageal endoscopy revealed multiple whitish punctate lesions of sizes varying between 0.2 and 0.5 cm and predominantly middle‐third esophageal distribution. Endoscopic diagnosis was nonerosive GERD. Esophageal manometry and pH monitoring are requested, but the patient does not attend the test. Histopathological examination showed esophageal squamous epithelium containing isolated sebaceous glands throughout (Figs 1 and 2).

## Discussion

Sebaceous glands are very rarely found in the esophagus. The largest series shows an incidence of 0.00465% and a 0.41% annual occurrence 2. A literature search found 38 articles in English, thirteen of which involve 22 cases with varying information that enables partial comparison 1, 2, 3, 4, 5, 6, 7, 8, 9, 10, 11, 12, 13 [Table 1]. Additionally, it includes our case report.

**Table 1:** Summary of clinical and endoscopic findings of patients with heterotopic sebaceous glands in the esophagus

Age	Sex	Number of lesions	Location	Size (mm)	Symptoms	Monitoring (month)	References
52	M	1	Middle third	2	Asymptomatic	No data	1
45	F	More than 100	Lower third	1–2	Asymptomatic	No data	1
69	F	More than 100	Lower third	1–2	Asymptomatic	No data	1
46	M	No data	No data	No data	Peptic	No data	2
71	M	No data	No data	No data	Asymptomatic	No data	2
60	M	No data	No data	No data	Peptic	No data	2
65	M	No data	No data	No data	Asymptomatic	No data	2
49	F	No data	No data	No data	Asymptomatic	No data	2
55	F	No data	No data	No data	Asymptomatic	No data	2
56	M	More than 100	Middle and lower third	1–20	Reflux	No data	3
50	F	More than 100	The entire esophagus	No data	Reflux	No data	4
65	M	20	Middle and lower third	2–4	Peptic	No data	5
47	M	1	Middle third	4	Peptic	No data	6
85	M	Multiple	Upper third	3–4	Reflux	20	7
58	F	Multiple	Middle and lower third	2–6	Reflux	20	7
39	F	1	Middle third	5	Peptic	4	7
45	F	More than 100	No data	No data	Peptic	No data	8
44	F	Multiple	No data	No data	Reflux	No data	9
53	F	More than 100	Middle and lower third	5	Asymptomatic	No data	10
50	M	Multiple	The entire esophagus	No data	Reflux	No data	11
51	M	No data	No data	No data	Peptic	No data	12
54	M	No data	No data	No data	Reflux	No data	13
49	M	Multiple	Middle third	2–5	Reflux	No data	Case report

There is a slight predominance in males, with ages ranging from 39 to 69 years and an average age of 55 years. The majority of patients were symptomatic, predominantly with GERD, while a minority had nonspecific peptic symptoms. Endoscopic studies reported single and multiple lesions [greater than one hundred, while other studies did not specify the number of lesions]. Endoscopic features include punctate lesions and yellowish plaque lesions, and the main endoscopic differential diagnoses comprise glycogenic acanthosis, candidiasis, xanthomas, and papillomas. Monitoring did not turn up any differences in the evolution of these patients 2, 3, 4, 5, 6, 7, 8, 9, 10, 11, 12, 13.

The sebaceous glands are of ectodermal origin and associated with hair follicles. They are numerous in facial skin, on the buccal mucosa, the vermillion of the lip [Fordyce spots], prepuce, labia minora, and the parotid gland. Histologic recognition is easy; these structures are composed of one or more lobular acini whose periphery contains germ, cubic, and flat cells with visible nucleoli and basophilic cytoplasm with lipid droplets. As the cytoplasm matures, it forms lipid vacuoles and the more mature cells disintegrate, discharging cellular debris [sebum] into the excretory duct. This duct empties into the hair follicle infundibulum. The excretory duct has several lobes lined with keratinized squamous epithelium 14. Finding sebaceous glands in the esophagus is controversial. A heterotopic histogenesis, in question due to the endodermal embryonic origin of the esophagus [as opposed to the ectodermal origin of the sebaceous glands], has been proposed. Another theory of histogenesis proposes sebaceous metaplasia arising in patients with a history of GERD. 14.

## Conclusion

This case report describes the exceptional discovery of esophageal sebaceous glands in a patient with GERD. Proper identification ruled out any reflux‐associated pathology. Despite the extreme rarity of this entity, it is considered a lesion with no malignant potential. The innocuous nature of this finding did not alter routine GERD monitoring.

## Consent

The patient's informed consent was obtained in writing for the publication of this case and the accompanying pictures. A copy of this written consent is available for review by the magazine's editor‐in‐chief.

## Authorship

All those involved have read and approved the final manuscript. NM: performed the histopathological examination and conceived and took part in the report's design and discussion. VT: did the literature review, design, and discussion. HP: provided the patient's clinical information, monitoring, and endoscopic examination. LR: took part in the report's design and discussion.

## Conflict of Interest

The authors state they have no conflict of interest.
